# Efficacy of lymph node dissection by node zones according to tumor location for esophageal squamous cell carcinoma

**DOI:** 10.1007/s10388-015-0515-3

**Published:** 2015-11-17

**Authors:** Yuji Tachimori, Soji Ozawa, Hodaka Numasaki, Hisahiro Matsubara, Masayuki Shinoda, Yasushi Toh, Harushi Udagawa, Mitsuhiro Fujishiro, Tsuneo Oyama, Takashi Uno, The Registration Committee for Esophageal Cancer of the Japan Esophageal Society

**Affiliations:** Division of Esophageal Surgery, National Cancer Center Hospital, 5-1-1 Tsukiji, Chuo-ku, Tokyo, 104-0045 Japan; Department of Gastroenterological Surgery, Tokai University School of Medicine, Isehara, Japan; Department of Medical Physics and Engineering, Osaka University Graduate School of Medicine, Osaka, Japan; Department of Frontier Surgery, Chiba University Graduate School of Medicine, Chiba, Japan; Department of Gastroenterological Surgery, Aichi Cancer Center Hospital, Nagoya, Japan; Department of Gastroenterological Surgery, Kyushu Cancer Center, Fukuoka, Japan; Department of Gastroenterological Surgery, Toranomon Hospital, Tokyo, Japan; Department of Endoscopy and Endoscopic Surgery, Graduate School of Medicine, University of Tokyo, Tokyo, Japan; Department of Gastroenterology, Saku General Hospital, Nagano, Japan; Department of Radiology, Graduate School of Medicine, Chiba University, Chiba, Japan

**Keywords:** Esophageal cancer, Squamous cell carcinoma, Lymphadenectomy, Metastasis, Survival

## Abstract

**Background:**

The extent of node dissection in esophageal cancer surgery is usually estimated by the number of resected nodes, irrespective of the area of dissection. The efficacy of lymph node dissection by area was evaluated according to the location of the primary tumor.

**Methods:**

The study group comprised the 3827 patients who underwent R0 esophagectomy with three-field lymph node dissection for squamous cell carcinoma, registered in a nationwide registry in Japan. The areas of lymph node were classified into zones according to AJCC Staging Manual. The Efficacy Index (EI) calculating the frequency and patient survival of metastases to each zone was investigated according to tumor location.

**Results:**

The EI was high in supraclavicular and upper mediastinal zones in patients with upper esophageal tumors, highest in upper mediastinal zone followed by supraclavicular and perigastric zones in patients with middle esophageal tumors, and highest in perigastric zone followed by upper and lower mediastinal zones in patients with lower esophageal tumors. In patients with middle and lower esophageal cT1 tumors, the EIs of upper mediastinal and perigastric zones were higher than middle and lower mediastinal zones.

**Conclusion:**

The EIs of each zone were differed by tumor location. The extent of lymph node dissection should be estimated by the dissected zones and modified by the tumor location. Supraclavicular dissection is indispensable for patients with upper esophageal tumors, and recommended for patients with middle esophageal tumors. Upper mediastinal dissection is recommended for all patients with thoracic esophageal squamous cell carcinoma, irrespective of the location.

## Introduction

Despite recent advances in multidisciplinary approaches, surgical resection remains the standard treatment for potentially resectable esophageal carcinoma. In addition to primary tumor resection, removal of all potentially involved lymph nodes is essential for achieving cure. In the present 7th UICC TNM classification [1] and the 7th AJCC Cancer Staging manual [2], the regional nodes are not varied irrespective of the location of the primary tumor. The extent of lymph node dissection in esophageal cancer surgery is estimated by the number of resected regional lymph nodes, irrespective of the area of dissection [2]. However, many surgeons accept that the area of nodal dissection should be modified according to the location of the primary tumor in an individual patient.

The purpose of this retrospective study was to evaluate the efficacy of lymph node dissection by the area based on the location of the primary tumor, calculating the frequency and patient survival of metastases to the area in patients with thoracic esophageal squamous cell carcinoma who underwent esophagectomy with curative intent. This study was based on a large, multi-institutional, nationwide registry of esophageal cancer maintained by the Japan Esophageal Society.

## Methods

### Patients

A comprehensive registry of esophageal cancer in Japan has been maintained by the Japan Esophageal Society since 1976. All patient data, including demographic characteristics, symptoms, clinical stage, treatment features, and survival information, were collected. Surgical features, clinical and pathological stage, and detailed lymph node metastatic status were also collected for patients who underwent surgery.

**Fig. 1:**
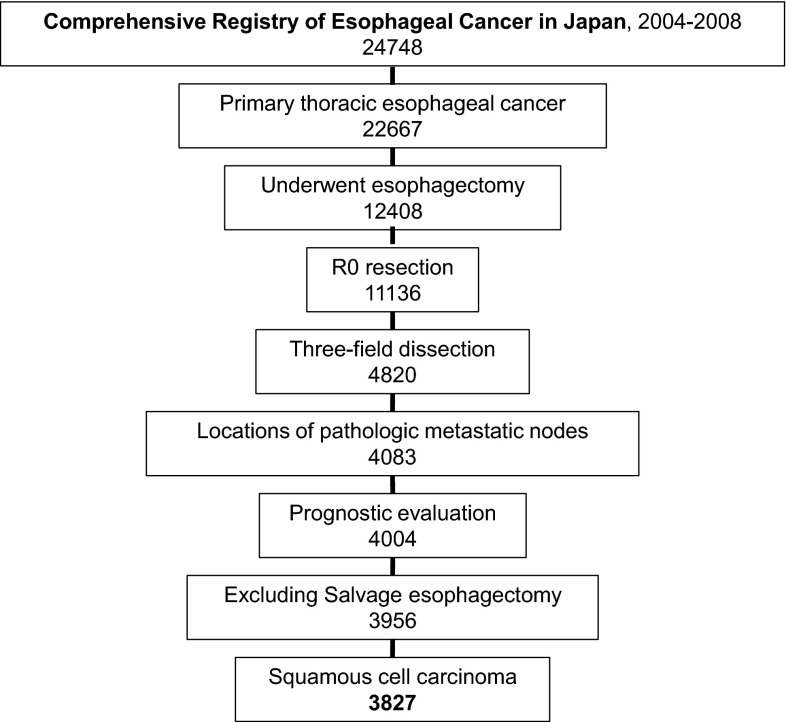
Patient disposition chart

A total of 24,748 patients with primary esophageal tumor treated in 2004, 2005 and 2006, and 2007 and 2008 were registered in 2011, 2012, and 2014, respectively, from 239 institutions in Japan [3–7]. Of the 24,748 patients, 22,667 had primary thoracic esophageal tumor, excluding cervical esophageal tumor and Siewert type II and type III esophagogastric junction cancers [8]. Of the 12,408 patients who underwent esophagectomy, 11,136 underwent R0 resection, and patients who underwent R1 and R2 resections were excluded due to limited node dissection. Of the 11,136 patients who underwent R0 resection, 4820 (43.3 %) patients underwent esophagectomy with three-field lymph node dissection [9, 10]. For the purpose of evaluating the frequency of metastasis to all regional node areas precisely, only the patients who underwent esophagectomy with three-field lymph node dissection were selected. The cervical, mediastinal, and abdominal lymph nodes were dissected. Dissections of supraclavicular nodes and cervical paraesophageal nodes were required for cervical dissection by three-field dissection in the registration. Since it was based on a multi-institutional, nationwide registry, the selection of patients and indications for three-field dissection depended on each institution and each surgeon, and were not specified. The three-field dissection was performed in 60.5 % of patients with upper esophageal tumor, 49.5 % of patients with middle esophageal tumor, and 30.8 % of patients with lower esophageal tumor. It was performed in 36.5 % of patients with cT1 tumor and 48.2 % of patients with cT2-4 tumor. Of the 4820 patients who underwent esophagectomy with three-field lymph node dissection for R0 resection, information about the locations of pathological metastatic lymph nodes was available for 4083 patients, and outcome evaluations were available in 4004 patients. Of the 3956 patients excluding 48 patients who received definitive chemoradiotherapy and underwent salvage esophagectomy, 3827 patients (97 %) had squamous cell carcinoma including adenosquamous carcinoma and basaloid squamous carcinoma, 64 patients (1.6 %) had adenocarcinoma, and 65 patients had other tumors including undifferentiated tumor, carcinosarcoma and malignant melanoma. The total study group comprised 3827 patients who underwent R0 resection and esophagectomy with three-field lymph node dissection for squamous cell carcinoma from 155 institutions (Fig. 1).

### Tumor classification

Clinical stages for all patients were recorded according to the 6th edition of the UICC TNM Classification [11]. Pathological stages for all patients were re-assessed according to the 7th edition of the UICC TNM Classification [1]. The thoracic esophagus was divided into three anatomical subsites: upper, middle, and lower. The tumor location is regarded as the point of deepest tumor invasion according to the Japanese Classification [12], which in clinical practice is the epicenter of the tumor.

The areas of lymph node metastasis were recorded according to the lymph node stations adopted by the Japanese Classification [12]. There are some differences in the definition of lymph node stations between the Japanese Classification [12] and AJCC Staging Manual [2] (Table 1). This difference in the anatomical definition of each lymph node station might have influenced the nodal categorization. However, with the database collected, there was no way to reasonably reconcile or amend such differences. So, lymph node stations were classified into lymph node zones according to the map in AJCC Staging Manual [2] (Table 1). The middle mediastinal zone and the lower mediastinal zone were divided by caudal margin of the inferior pulmonary vein.

**Table 1:** Node zones

Node zone	Station number (JES)	Name of node station (JES)	Station number (AJCC)	Name of node station (AJCC)
Supraclavicular	104R	Right supraclavicular	1	Supraclavicular
	104L	Right supraclavicular	1	Supraclavicular
	101R	Right cervical paraesophageal		(Cervical paraesophageal)
	101L	Right cervical paraesophageal		(Cervical paraesophageal)
Upper mediastinal	105	Upper paraesophageal	3p	Posterior mediastinal
	106pre	Pretracheal	2R	Right upper paratracheal
	106recR	Right recurrent nerve	2R	Right upper paratracheal
	106recL	Right recurrent nerve	2L	Left upper paratracheal
	106tbR	Right tracheobronchial	4R	Right lower paratracheal
	106tbL	Right tracheobronchial	4L	Left lower paratracheal
Middle mediastinal	107	Subcarinal	7	Subcarinal
	108	Middle paraesophageal	8 m	Middle paraesophageal
	109R	Right main bronchus	10R	Right tracheobronchial
	109L	Left main bronchus	10L	Left tracheobronchial
Lower mediastinal	110	Lower paraesophageal	8 l	Lower paraesophageal
	111	Supradiaphragmatic	15	Diaphragmatic
	112	Posterior mediastinum	9	Pulmonary ligament
Perigastric	1	Right cardiac	16	Paracardial
	2	Left cardiac	16	Paracardial
	3	Lesser curvature		
	7	Left gastric artery	17	Left gastric artery
Celiac	9	Celiac	20	Celiac
	8	Common hepatic artery	18	Common hepatic
	11	Splenic artery	19	Splenic
	19	Infradiaphragmatic		

### Method of analysis

To evaluate the efficacy of nodal dissection at each zone, the efficacy index (EI) was calculated by multiplying the frequency (%) of metastases to a zone and the 5-year survival rate (%) of patients with metastases to that zone, and then dividing by 100 [13–15]. The EI was investigated according to tumor location. The EI was also determined by clinical T factor: cT1 and cT2-4. Survival rates were constructed using the Kaplan–Meier method. Statistical analysis was performed using the SPSS Statistics Software Package (SPSS Inc., Chicago, IL, USA).

## Results

Patient characteristics and findings are listed in Table 2. The location of the tumors was the upper esophagus in 629 patients (16.4 %), the middle esophagus in 2215 (57.9 %), and the lower esophagus in 983 (25.7 %).

**Table 2:** Patients’ characteristics and tumor findings

Characteristic or finding	No. (%)
Median age (range), year	63.0 (30–85)
Sex	
Male	3293 (86.0 %)
Female	534 (14.0 %)
Tumor location	
Upper	983 (16.4 %)
Middle	2215 (57.9 %)
Lower	629 (25.7 %)
Preoperative therapy	
Chemotherapy	515 (13.5 %)
Chemoradiotherapy	238 (6.2 %)
Radiotherapy	3 (0.1 %)
None	3071 (80.2 %)
Clinical T classification	
T1	1160 (30.3 %)
T2	701 (18.3 %)
T3	1810 (47.3 %)
T4	156 (4.1 %)
Pathologic positive node number (including supraclavicular node)	
N0	1616 (42.2 %)
N(1–2)	843 (22.0 %)
N(3–6)	903 (23.6 %)
N(7–)	465 (12.2 %)

Preoperative neoadjuvant chemoradiotherapy was administered to 238 patients (6.2 %), 515 patients (13.5 %) received preoperative chemotherapy, and 3 patients (0.1 %) received preoperative radiotherapy. Preoperative therapy was under clinical study [16] and not standard for esophageal cancer in Japan during the registration period. The selection of patients, indications, and therapeutic approach to preoperative therapy depended on each institution and were not specified.

The 30-day operative mortality rate was 0.9 % (33 patients) and 90-day mortality was 1.8 % (69 patients). The 5-year survival rate for all patients was 57.5 %.

The frequency of metastasis, the 5-year survival rate of patients with metastases, and the EI of each zone are presented according to tumor location in Table 3. The frequency of metastasis and the EI of each zone were different by tumor locations. In patients with upper esophageal tumors, the EIs of the supraclavicular zone and the upper mediastinal zone were high. In contrast, those of the middle mediastinal, lower mediastinal and perigastric zones were low. In patients with middle esophageal tumors, the EI of the upper mediastinal zone was the highest, followed by those of supraclavicular zone and perigastric zones. In patients with lower esophageal tumors, the EI of perigastric zone was the highest, followed by those of upper mediastinal and lower mediastinal zones. The EIs of celiac zone were the lowest among all the zones in patients with thoracic squamous cell carcinoma. Differences of the EIs between zones mostly depended on difference of the frequency of metastasis to zones. Differences of the 5-year survival rates of patients with metastases between zones were less.

**Table 3:** The frequency of metastasis, the 5-year survival rate of patients with metastases, and the EI of each zone according to tumor location for esophageal squamous cell carcinoma

Lymph node zone	Upper esophageal cancer			*n* = 629	Mid esophageal cancer			*n* = 2215	Lower esophageal cancer			*n* = 983
	Positive patients	Positive rate (%)	5-year survival rate (%)	Efficacy index	Positive patients	Positive rate (%)	5-year survival rate (%)	Efficacy index	Positive patients	Positive rate (%)	5-year survival rate (%)	Efficacy index
Supraclavicular zone	210	33.4	42.3	14.1	505	22.8	40.5	9.2	173	17.6	30.0	5.3
Upper mediastinal zone	270	42.9	41.1	17.6	829	37.4	40.0	15.0	249	25.3	32.6	8.2
Middle mediastinal zone	59	9.4	32.2	3.0	462	20.9	29.0	6.1	193	19.6	24.1	4.7
Lower mediastinal zone	27	4.3	33.1	1.4	254	11.5	33.5	3.9	242	24.6	34.2	8.4
Perigastric zone	62	9.9	31.1	3.1	618	27.9	33.2	9.3	479	48.7	36.5	17.8
Celiac zone	5	0.8	0.0	0.0	89	4.0	26.1	1.0	104	10.6	27.0	2.9

The frequency of metastasis, the 5-year survival rate of patients with metastases, and the EIs of each zone in patients with cT1 tumor are presented in Table 4. In patients with upper esophageal cT1 tumors, the EI of the upper mediastinal zone was highest. However, in patients with middle and lower esophageal cT1 tumors, the EIs of the middle and lower mediastinal zones were lower than those of the upper mediastinal and perigastric zones. In 22 patients with lower esophageal cT1 tumors and metastasis to the supraclavicular zone, 9 patients had the proximal margin of the tumor in the middle esophagus. In 27 patients with lower esophageal cT1 tumors and metastasis to the upper mediastinal zone, 14 patients had the proximal margin of the tumor in the middle esophagus.

**Table 4:** The frequency of metastasis, the 5-year survival rate of patients with metastases, and the EI of each zone according to tumor location for cT1 esophageal squamous cell carcinoma

Lymph node zone	Upper esophageal cancer			*n* = 211	Mid esophageal cancer			*n* = 752	Lower esophageal cancer			*n* = 197
	Positive patients	Positive rate (%)	5-year survival rate (%)	Efficacy index	Positive patients	Positive rate (%)	5-year survival rate (%)	Efficacy index	Positive patients	Positive rate (%)	5-year survival rate (%)	Efficacy index
Supraclavicular zone	42	19.9	60.7	12.1	94	12.5	58.9	7.4	22	11.2	39.4	4.4
Upper mediastinal zone	56	26.5	62.8	16.6	161	21.4	57.5	12.3	27	13.7	58.2	8.0
Middle mediastinal zone	2	0.9	50.0	0.5	32	4.3	34.4	1.5	12	6.1	22.2	1.4
Lower mediastinal zone	2	0.9	0.0	0.0	30	4.0	66.9	2.7	17	8.6	46.3	4.0
Perigastric zone	8	3.8	15.0	0.6	76	10.1	53.9	5.4	34	17.3	45.2	7.8
Celiac zone	0	0.0			11	1.5	36.4	0.5	5	2.5		

The frequency of metastasis, the 5-year survival rate of patients with metastases, and the EIs of each zone in patients with cT2-4 tumors are presented in Table 5. In patients with middle esophageal cT2-4 tumors, frequency of lymph node metastasis and the EI of the middle mediastinal zone was increased dramatically compared with patients with cT1 tumors, but still lower than those of the upper mediastinal and perigastric zones. In patients with lower esophageal cT2-4 tumors, the EI of the upper mediastinal zones was as high as that of the lower mediastinal zones.

**Table 5:** The frequency of metastasis, the 5-year survival rate of patients with metastases, and the EI of each zone according to tumor location for cT2-4 esophageal squamous cell carcinoma

Lymph node zone	Upper esophageal cancer			*n* = 418	Mid esophageal cancer			n = 1146	Lower esophageal cancer			n = 786
	Positive patients	Positive rate (%)	5-year survival rate (%)	Efficacy index	Positive patients	Positive rate (%)	5-year survival rate (%)	Efficacy index	Positive patients	Positive rate (%)	5-year survival rate (%)	Efficacy index
Supraclavicular zone	168	40.2	37.8	15.2	411	28.1	36.6	10.3	151	19.2	27.7	5.3
Upper mediastinal zone	214	51.2	34.6	17.7	668	45.7	36.2	16.5	222	28.2	27.8	7.8
Middle mediastinal zone	57	13.6	31.8	4.3	430	29.4	28.4	8.3	181	23.0	23.8	5.5
Lower mediastinal zone	25	6.0	34.5	2.1	224	15.3	28.9	4.4	225	28.6	33.2	9.5
Perigastric zone	54	12.9	33.8	4.4	542	37.0	30.3	11.2	445	56.6	35.7	20.2
Celiac zone	5	1.2	0.0	0.0	78	5.3	24.6	1.3	99	12.6	25.3	3.2

## Discussion

The present study showed that the efficacies of node dissection differed by zone of lymph node. Many previous studies demonstrated that the number of lymph nodes removed is an independent predictor of survival after esophagectomy for cancer [17–22]. The extent of lymph node dissection in esophageal cancer surgery was estimated by the number of resected regional lymph nodes. In the present 7th UICC TNM Classification, it is recommended that histological examination of a regional lymphadenectomy specimen ordinarily include 7 or more lymph nodes [1]. The 7th AJCC staging manual recommends that, for pT1, approximately 10 nodes must be resected to maximize survival; for pT2, 20 nodes; and for pT3 or pT4, 30 nodes or more [2], based on the data of the worldwide esophageal cancer collaboration [22]. In NCCN guideline, in patients undergoing esophagectomy without induction chemoradiation, at least 15 lymph nodes should be removed to achieve adequate nodal staging [23]. However, when only the node zones with low EI are dissected, and those with high EI are not dissected, the efficacy of node dissection is low, even more than 20 nodes are dissected. Thus, the effective extent of node dissection should be modified by the EIs of node zones.

EIs of each node zone were differed by tumor location. The zones for dissection should be modified according to the location of the tumor. For upper esophageal tumors, the upper mediastinal zone had the highest EI and is the most important dissection target. The EI of supraclavicular zone was also high and supraclavicular node dissection is indispensable for patients with upper esophageal tumor. Supraclavicular nodes should be classified as regional nodes for tumors in the upper esophagus. In patients with tumor in the middle esophagus, upper mediastinal zone had the highest EI followed by perigastric and supraclavicular zones. For patients with tumor in the middle esophagus, the most common type of esophageal tumor in Asia, not only mediastinal and abdominal, but also cervical dissection by the three-field approach is recommended. Patients with tumor in the lower esophagus had the highest EI in perigastric zone. However, the EI of upper mediastinal zone was as high as that of lower mediastinal zone. Upper mediastinal dissection is recommended for all patients with thoracic esophageal squamous cell carcinoma, irrespective of the location.

The present study showed that the frequency of metastasis and the EI did not reflect the anatomical distance from the primary tumor, but rather the lymphatic drainage system reported previously [24, 25]. Even with tumors located in the middle and lower esophagus, lymphatic metastasis was frequent in the upper mediastinal and perigastric zones. The conventional hypothesis is that tumor cells involve the nearby nodes first, then spread to nodes a little further, and finally reach distant nodes. The extent of node dissection has been estimated by anatomical distance from the primary tumor to the dissected node area. However, in patients with middle and lower esophageal cT1 tumors, the EIs of the middle and lower mediastinal zone were lower than those of upper mediastinal zone and perigastric zone. Therefor extent of dissection in patients with cT1 tumors should be not tailored according to the anatomical distance from the tumor, but according to the EI.

Many patients with lower esophageal cT1 tumors and the proximal margin of the tumor in the middle esophagus had metastasis to the supraclavicular zone and the upper mediastinal zone. It suggests that the proximal nodal spread to the supraclavicular and upper mediastinal nodes is reflect to the location of proximal margin of the tumor. The attention to the proximal margin of tumor should be paid in planning the extend of node dissection. The proximal margin of squamous cell carcinoma tends to be more proximal than those of adenocarcinoma. Supraclavicular and upper mediastinal node metastasis are not neglected.

In this study, lymph node stations were classified into lymph node zones according to the map in AJCC Staging Manual. In surgical dissection and in identification and labeling during pathological examination of specific lymph node, and also in planning of irradiation field, lymph node zones are more practical than small neighboring lymph node stations.

The present study was based on patients with squamous cell carcinoma, and patients with adenocarcinoma were not included. However, in Asian patients, including Japanese patients, squamous cell carcinoma remains the predominant histological cell type of esophageal cancer, and more than half of tumors locates in the upper and middle esophagus.

In conclusion, the EIs of each zone were differed by tumor location. The extent of lymph node dissection should be estimated by the dissected lymph node zones and modified by the tumor location. Supraclavicular dissection is indispensable for patients with upper esophageal tumors and recommended for patients with middle esophageal tumors. Upper mediastinal dissection is recommended for all patients with thoracic esophageal squamous cell carcinoma, irrespective of the location.

## References

[1] Sobin LH, Gospodarowicz MK, Wittekind C. UICC International Union Against Cancer. TNM classification of malignant tumors. 7th ed. Wiley-Blackwell, New York, NY 2009.

[2] Edge SB, Byrd DR, Compton CC, et.al. American Joint Committee on Cancer. AJCC Cancer Staging Manual. 7th ed. Springer, New York, NY 2010. 10.1245/s10434-010-0985-4 20180029

[3] Ozawa S, Tachimori Y, Baba H (2012). Comprehensive Registry of Esophageal Cancer in Japan, 2004. Esophagus.

[4] Tachimori Y, Ozawa S, Fujishiro M (2014). Comprehensive Registry of Esophageal Cancer in Japan, 2005. Esophagus.

[5] Tachimori Y, Ozawa S, Fujishiro M (2014). Comprehensive Registry of Esophageal Cancer in Japan, 2006. Esophagus.

[6] Tachimori Y, Ozawa S, Numasaki H (2015). Comprehensive Registry of Esophageal Cancer in Japan, 2007. Esophagus.

[7] Tachimori Y, Ozawa S, Numasaki H (2015). Comprehensive Registry of Esophageal Cancer in Japan, 2007. Esophagus.

[8] Siewert JR, Stein HJ (1996). Adenocarcinoma of the gastroesophageal junction: classification, pathology and extent of resection. Dis Esophagus.

[9] Kato H, Watanabe H, Tachimori Y (1991). Evaluation of neck lymph node dissection for thoracic esophageal carcinoma. Ann Thorac Surg.

[10] Akiyama H, Tsurumaru M, Udagawa H (1994). Radical lymph node dissection for cancer of the thoracic esophagus. Ann Surg.

[11] Sobin LH, Wittekind C. UICC International Union Against Cancer. TNM classification of malignant tumors. 6th ed. Wiley-Liss, New York, NY 2002.

[12] Japan Esophageal Society (2009). Japanese Classification of Esophageal Cancer, 10th edition: part I. Esophagus.

[13] Sasako M, McCulloch P, Kinoshita T (1995). New method to evaluate the therapeutic value of lymph node dissection for gastric cancer. Br J Surg.

[14] Fujita H, Aikou T, Tsurumaru M (2007). A new N category for cancer in the esophagogastric junction based on lymph node compartments. Esophagus.

[15] Udagawa H, Ueno M, Shinohara H (2012). The importance of grouping of lymph node stations and rationale of three-field lymphoadenectomy for thoracic esophageal cancer. J Surg Oncol.

[16] Ando N, Kato H, Igaki H (2012). A randomized trial comparing postoperative adjuvant chemotherapy with cisplatin and 5-fluorouracil versus preoperative chemotherapy for localized advanced squamous cell carcinoma of the thoracic esophagus (JCOG9907). Ann Surg Oncol.

[17] Kang CH, Kim YT, Jeon SH (2007). Lymphadenectomy extent is closely related to long-term survival in esophageal cancer. Eur J Cardiothorac Surg.

[18] Schwarz RE, Smith DD. Clinical impact of lymphadenectomy extent in resectable esophageal cancer. J Gastrointest Surg 2007;11:1384–93; discussion 1393–4. 10.1007/s11605-007-0264-2 17764019

[19] Altorki NK, Zhou XK, Stiles B (2008). Total number of resected lymph nodes predicts survival in esophageal cancer. Ann Surg.

[20] Greenstein AJ, Litle VR, Swanson SJ (2008). Effect of the number of lymph nodes sampled on postoperative survival of lymph node-negative esophageal cancer. Cancer.

[21] Peyre CG, Hagen JA, DeMeester SR (2008). The number of lymph nodes removed predicts survival in esophageal cancer: an international study on the impact of extent of surgical resection. Ann Surg.

[22] Rizk NP, Ishwaran H, Rice TW (2010). Optimum lymphadenectomy for esophageal cancer. Ann Surg.

[23] Ajani JA, D’Amico TA, Almhanna K (2015). Esophageal and esophagogastric junction cancers, version 1.2015. J Natl Compr Cancer Netw.

[24] Kuge K, Murakami G, Mizobuchi S (2003). Submucosal territory of the direct lymphatic drainage system to the thoracic duct in the human esophagus. J Thorac Cardiovasc Surg.

[25] Tachimori Y, Nagai Y, Kanamori N (2011). Pattern of lymph node metastases of esophageal squamous cell carcinoma based on the anatomical lymphatic drainage system. Dis Esophagus.

